# Novel Bioengineering Strategies to Improve Bioavailability and *In Vivo* Circulation of H-Ferritin Nanocages by Surface Functionalization

**DOI:** 10.1021/acsomega.2c07794

**Published:** 2023-02-17

**Authors:** Marta Sevieri, Mattia Pinori, Arianna Chesi, Arianna Bonizzi, Leopoldo Sitia, Marta Truffi, Carlo Morasso, Fabio Corsi, Serena Mazzucchelli

**Affiliations:** ‡ Nanomedicine Laboratory, Department of Biomedical and Clinical Sciences, Università degli Studi di Milano, 20157 Milan, Italy; † Nanomedicine and Molecular Imaging Lab, Istituti Clinici Scientifici Maugeri IRCCS, 27100 Pavia, Italy; ⊥ Breast Unit, Istituti Clinici Scientifici Maugeri IRCCS, 27100 Pavia, Italy

## Abstract

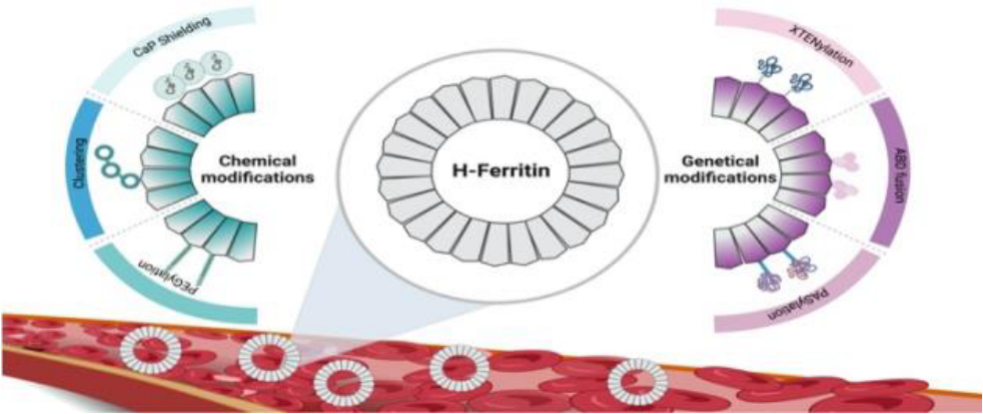

Due to its unique architecture and innate capability to specifically target cancer cells, ferritin has emerged as an attractive class of biomaterials for drug delivery. In many studies, various chemotherapeutics have been loaded into ferritin nanocages constituted by H-chains of ferritin (HFn), and their related anti-tumor efficacy has been explored by employing different strategies. Despite the multiple advantages and the versatility of HFn-based nanocages, there are still many challenges to face for their reliable implementation as drug nanocarriers in the process of clinical translation. This review aims at providing an overview of the significant efforts expended during recent years to maximize the features of HFn in terms of increased stability and *in vivo* circulation. The most considerable modification strategies explored to improve bioavailability and pharmacokinetics profiles of HFn-based nanosystems will be discussed herein.

## 1 Introduction

Over the past 20 years, there has been a growing interest in the study of nanomedicine due to its potential to address some of the issues of traditional drug delivery therapy in oncology.^1^ Although in preclinical settings many strategies based on nanoparticles demonstrate efficacy in tackling tumor growth and expanding survival, they unfortunately translate into a relatively low number of nanoformulations from which patients can effectively benefit.^2^

Considering that the success rate of clinical translation of many nanotechnological solutions, often too complicated, remains relatively low, the strategy of taking advantage of simpler nanomedicine platforms could be a winning choice. Indeed, leveraging biocompatible nanostrategies presenting high bloodstream stability and natural tumor homing could tackle safety issues and the translational challenges of traditional nanomedicines, closing the gap for real clinical applications. In this context, nanocages made from ferritin have attracted considerable attention in the biomedical and bioengineering fields by virtue of their unique features.^3^

Ferritin (Fn) is one of the most studied proteins and in nature is responsible first for the maintenance of intracellular iron concentrations and also for protection from oxidative stress.^3^ Human Fn is a globular multimeric protein made from 24 subunits of H (heavy) and L (light) Fn chains which can self-assemble in a cave-sphere quaternary structure, forming a stable nanocage of 12 nm diameter with an internal cavity 8 nm in diameter.^3^ Moreover, the salt bridges and hydrogen bonds that link subunits allow Fn to tolerate pH and temperature variations. This protein remains stable also in the presence of denaturing agents, which enables Fn to disassemble in extremely acidic (pH 2–3) or basic (pH 10–12) conditions and reassemble in a shape-memory manner when the pH returns to neutrality.^4^

By taking advantage from these unique structural and physicochemical features, nanotechnologists have exploited nanocages constituted only by H-chains of Fn (HFn) for drug delivery, accomplishing the loading of different types of compounds, like chemotherapeutics or fluorescent dyes.^5^ More importantly, the ability to directly target the human transferrin receptor 1 (TfR1), which is overexpressed in several cancer types, increases the attractiveness of HFn in the context of cancer treatment.^3^

## 2 H-Ferritin Nanocages for Drug Delivery

The applications of HFn in the biomedical field are multiple. Referring to the nanotechnology field, it can be exploited as a reaction chamber to produce metal or semiconductor nanoparticles (NPs). Indeed, the unique cavity of this type of nanocages is extensively exploited for the biomineralization of metal oxides (iron, manganese, cobalt, chromium, and nickel) to assemble semiconductor inorganic NPs with interesting fluorescent properties related to their size and shape.^3^ In addition to that, cancer treatment and vaccines development are two emerging dominant fields where HFn can be applied. In particular, the nanomedicine scenario has developed fast due to the global need for new therapeutic approaches and technologies against the ongoing pandemic of coronavirus disease 2019 (COVID-19).

An example is the nanoparticle vaccine based on ferritin designed against the SARS-CoV-2 Omicron variant. The ferritin structure was exploited to incorporate in the N-terminal position a protein A tag as a structural scaffold. Then, as an immunogen, the receptor binding domain (RBD) of SARS-CoV-2 Omicron spike protein was fused with an Fc tag at the C-terminus. Once purified, the RBD was assembled onto nanoparticles by the interaction of Fc and the protein A tag. This is a new design strategy for vaccines which can enhance the neutralizing immune responses.^6^

To date, HFn has found wider application in the oncological field, where it is employed especially as a delivery system for the diagnosis and treatment of tumors. Within HFn it is possible to encapsulate different compounds and chemotherapeutic drugs for oncological therapy. Some examples of drugs that can be easily entrapped in the HFn shell are cisplatin, carboplatin, and desferrioxamine B, since they have an innate tendency to bind metals. HFn containing cisplatin has been extensively studied and demonstrated to be important in tumor treatment in different applications, including the study of the apoptotic process and the treatment of melanoma.^7^ To date, a plethora of therapeutic drugs, e.g., paclitaxel, curcumin, daunomycin, doxorubicin, epirubicin, etc., have been loaded into the inner cavity of ferritin nanocages.^8,9^

Among the HFn-based nanoformulates, one the most studied in the literature involves the encapsulation of doxorubicin (DOX), a cytotoxic drug broadly used in anti-cancer therapy. Indeed, it has been widely demonstrated that DOX nanoformulation in HFn is able to improve efficacy and accumulation to the tumor and, above all, is decisive in reducing its cardiotoxicity as well as the serious side effects associated with this type of treatment.^4,10,11^

Another important chemotherapeutic drug that can be encapsulated in HFn is paclitaxel (PTX), employed for the treatment of advanced ovarian cancer and AIDS-related Kaposi sarcoma. Unfortunately, the clinical applications of PTX are limited due to its poor solubility and the lack of targeting. Loading this drug into HFn makes it possible to overcome the lack of targeting and demonstrates higher therapeutic efficacy and decreased systemic toxicity *in vivo*.^12^

Encapsulation in HFn is also reported to stabilize lipophilic drugs like curcumin. Indeed, once loaded into HFn, curcumin is found to be more stable and bioavailable, which reduces the premature degradation that occurs when it is used in free form. Although *in vitro* studies have demonstrated an effect of HFn-curcumin in controlling the proliferative activity on tumor cells, the low solubility of this drug that leads to a poor encapsulation efficacy prevented it from proceeding to further *in vivo* assessments.^13^

HFn-based systems have been employed also as carriers of miRNA and/or siRNA, due to their ability to protect their cargo from nuclease activity and to achieve tumor-targeted delivery. These short noncoding RNA molecules related with tumor progression and/or resistance can be delivered into HFn in combination with other standard treatments.^14^

HFn nanocages hold promise to also promote diagnostic imaging tools. One example is the HFn formulations enclosing indocyanine green (ICG), a fluorescent dye widely used in clinics for different purposes (e.g., lymph node mapping). HFn-ICG nanocages are reported to address the issues of rapid degradation and lack of specificity related to ICG, providing a suitable nanotracer for fluorescence-guided detection of cancer tissues.^5^

## 3 Limitations of H-Ferritin Nanocages

As previously reported, HFn nanocages have been studied with outstanding results as delivery systems in terms of specific tumor recognition and increased activity with lower side effects.^10,11^ Properties such as high biocompatibility and good biodegradability put HFn ahead of conventional materials in clinical translation for imaging and drug delivery purposes. Unfortunately, despite their many undeniable benefits, HFn-based nanosystems also have important vulnerabilities.

First, it is necessary to consider the short plasma half-life after systemic injection displayed by HFn, which leads to poor accumulation at tumor sites.^4,15^ Indeed, according to the results obtained by Yin et al., the 2–3 h half-life of human HFn in circulation as obtained for an HFn/DOX formulation is unsatisfactory, considering that this circulation time is shorter than those of the majority of other drug nanocarriers due to its relatively small particle size.^16^ In light of this, many efforts have been made toward the exploration of functionalization strategies for a broader application of HFn and improvement in treatment outcomes. Second, often the drug-binding ability of HFn is not completely satisfactory. Overall, both the yields and stability of the HFn–drug complexes might not meet the necessary requirements for its potential pharmaceutical applications.^15^

**Figure 1:**
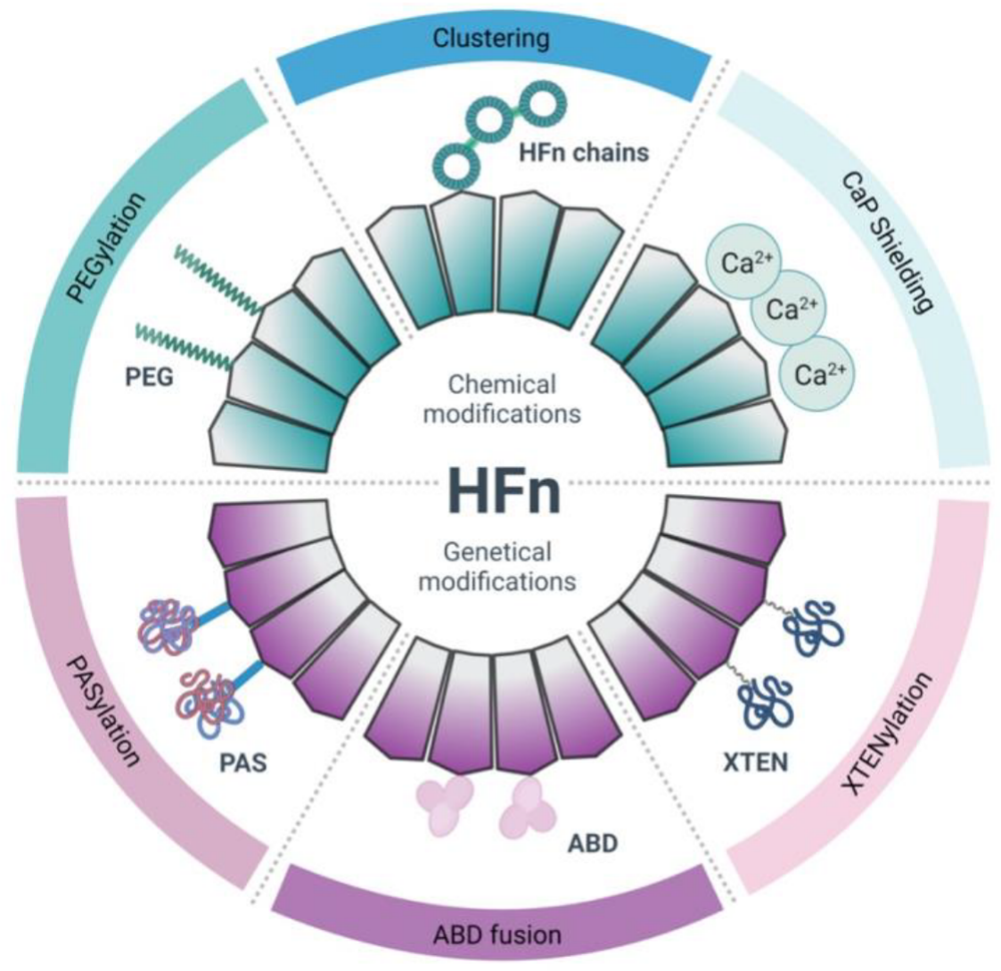
Scheme of chemical and genetical modifications of HFn aimed at improving stability and *in vivo* circulation.

In this review, we summarize actions proposed recently by researchers with the aim of addressing the short half-life that characterizes HFn to maximize its intrinsic capability to target specific tumor sites (Figure 1).

## 4 Chemical Modifications of H-Ferritin Nanocages

### 4.1 Conjugation of H-Ferritin Nanocages with Polyethylene Glycol (PEG) Molecules

It is worth noting that surface modifications of nanomaterials can strongly influence their performance as drug delivery vehicles by improving their biocompatibility, selectivity, and circulation in the bloodstream. Among the different approaches developed for the improvement of HFn’s features, multiple chemical modifications have been proposed, including conjugation with polyethylene glycol (PEG), reported to enhance the *in vivo* circulation time. Indeed, this strategy, named PEGylation, has been extensively employed to improve systemic circulation time and decrease immunogenicity, thus increasing the efficiency of drug and gene delivery to target cells and tissues.^17,18^ However, it has also been reported that PEGylation may interfere with the intrinsic ability of HFn to recognize TfR1 and target cancer cells.^19^ As a consequence, it has become necessary to introduce further adaptations to the classic PEGylation strategies.

An interesting PEG-masked HFn nanoplatform was developed for the treatment of melanoma. After performing a controlled modification of the HFn protein surface with a precise number of PEG molecules, the authors inserted a selective targeting moiety for melanoma cells, named α-MSH peptide. This targeting strategy was successful in improving the circulation half-life of HFn and achieving selective internalization by melanoma cells.^20^ However, it is worth noting that the PEGylation process presents several disadvantages, including increased costs and reduced yields.^15,21^ In addition, PEG, which has been considered non-antigenic for years, may be responsible for immunogenicity, as well as not being biodegradable, thus bringing possible issues in biosafety which cannot be neglected.^17^

### 4.2 Clustering of H-Ferritin Nanocages with PEG Molecules

Recently, an interesting strategy to precisely assemble nanostructures in a controlled manner has been proposed. More specifically, it uses a “bottom-up” hierarchical incorporation of protein building blocks in order to obtain highly ordered nanostructures by means of PEG chemical conjugation. In particular, the strategy of assembling more HFn via PEG chemical conjugation to achieve the multivalent binding of HFn, thus facilitating prolonged circulation time and accumulation within tumor cells, has been investigated. Two-armed PEG molecules were used to link free −NH_2_ groups of HFn in order to achieve nanostructured assemblies, named oligomeric nanozymes, composed by monomers, dimers, and multimers. After a detailed *in vitro* characterization, the behavior of different HFn nano-assemblies was evaluated in a murine model of colorectal cancer. It was observed that the assembly of four HFn nanocages displayed improved blood pharmacokinetics and circulation time compared to the mono- and bi-assemblies, as well as enhanced tumor uptake.^22^

### 4.3 Shielding of H-Ferritin Nanocages with Calcium Phosphate

**Figure 2:**
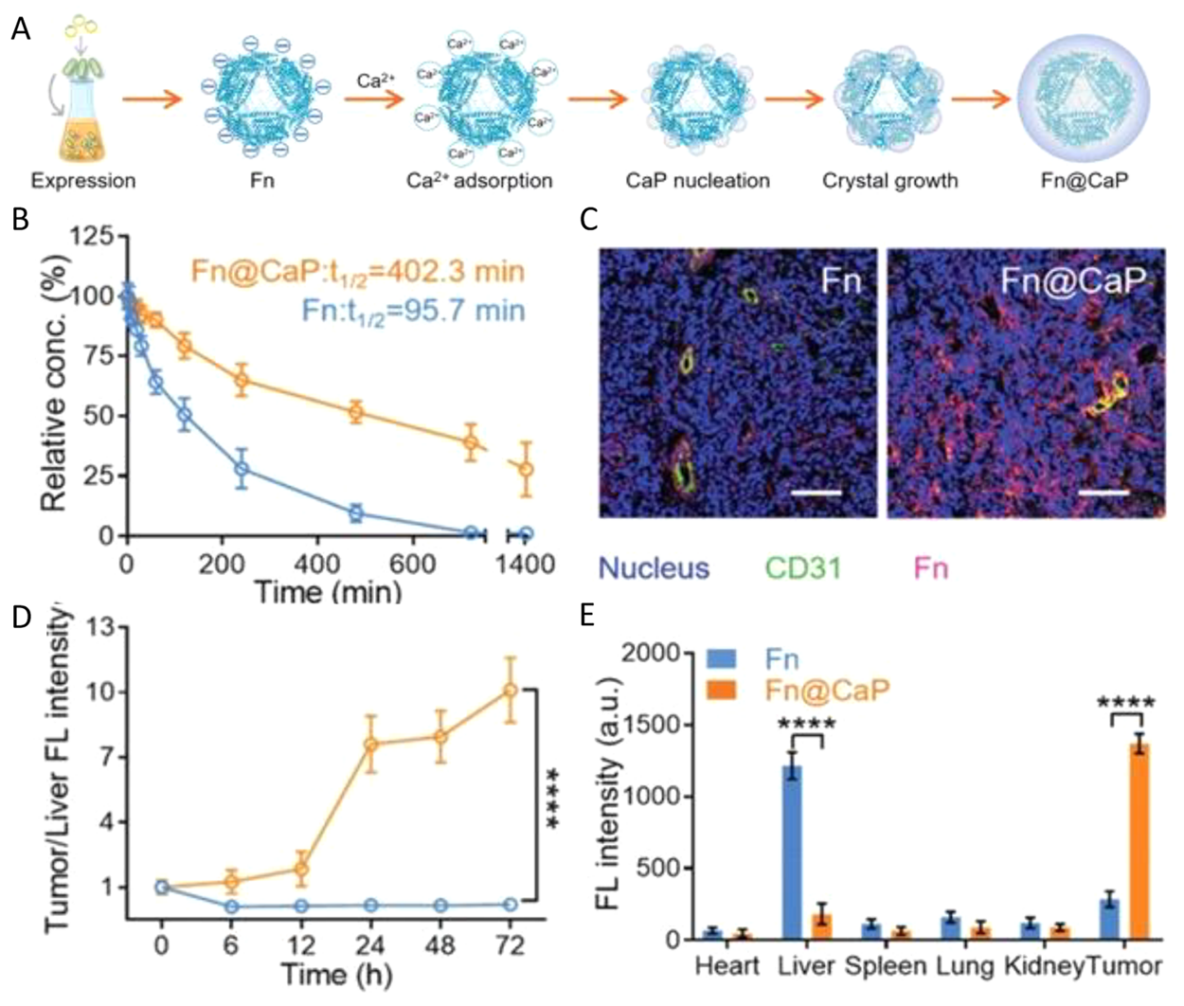
Fabrication and characterization of a biomineralized ferritin nanoplatform and evaluation of its *in vivo* distribution. (A) Schematic illustration of the preparation of the Fn@CaP nanoplatform. (B) Comparative analysis of blood half-life of Fn and Fn@CaP in HeLa tumor-bearing mice. (C) CLSM images of Fn and Fn@CaP accumulated in tumor tissues. (D) Comparative analysis of dynamic tumor versus liver fluorescence intensity of Cy7-labeled Fn and Fn@CaP in mice given the indicated treatments. (E) Comparative analysis of the fluorescence intensity of Cy7-labeled Fn and Fn@CaP at 24 h. Data represent the mean ± s.d. Statistical significance was calculated via a two-tailed Student’s *t* test (D, E). ***p < 0.001, ****p < 0.0001. Reproduced with permission from ref (23). Copyright 2021 Wiley-VCH GmbH.

It is known that the high expression of TfR1 in the liver may interfere with HFn accumulation in tumors. Indeed, it has been observed that a co-culture with liver cells may cause reduced uptake efficiency by tumor cells, thus negatively affecting HFn’s delivery to the tumor.^23^ To overcome this limitation, a biomineralization strategy of shielding HFn with a calcium phosphate (CaP) shell has been proposed. Indeed, the presence of a mineral-reinforced coating is expected to enhance HFn’s serum stability, as likewise observed for CaP-mineralized micelles.^24^ Wang et al. developed ferritin nanoparticles coated with CaP aimed at maintaining stability in the liver (pH 7.4) while re-exposing it in the weakly acidic tumor microenvironment (Figure 2A). This strategy is conceived therefore to protect the nanoparticle from hepatic TfR1 recognition and at the same time ensure selective dissolution of the CaP shell in the tumor to allow specific binding and uptake by tumor cells. Overall, the biomineralization method to encapsulate nanomaterials is beneficial for the improvement of the serum half-life of HFn in comparison to the nonmineralized control HFn (Figure 2B). In addition, mice administered with CaP-coated HFn nanocages showed a dramatic reduction of liver accumulation and a 12-fold larger efficiency of tumor-specific HFn delivery (Figure 2C–E). Ultimately, extensive evaluations performed on multiple cell lines and patient-derived xenograft models supported the relevance of this nanoplatforms as an efficient nanostrategy for promoting tumor targeting and accumulation (Figure 2).^23^

## 5 Genetical Modifications of H-Ferritin Nanocages

### 5.1 PASylation of H-Ferritin Nanocages

Over the years, genetic engineering techniques have been applied for the development of modified ferritin nanoparticles, as a complement to the chemical modifications previously discussed. Among the genetic modifications proposed, an important role is played by the modification known as PASylation.^25^ This modification, which is based on the genetic fusion of biopharmaceuticals such as proteins, peptides, and low-molecular-weight drugs with a sequence rich in proline (P), alanine (A), and serine (S), was designed by Schlapschy with the aim of mimicking PEG while gaining advantages in biocompatibility and biodegradability as well as in increasing circulation time.^16^

Moreover, PAS sequences show high solubility in physiological solution and adopt stable random coil conformations, leading to expanded hydrodynamic volumes. Consequently, PAS conjugates show retarded kidney filtration and prolonged pharmacokinetics *in vivo*.^26^

This strategy has been applied to HFn by genetically fusing PAS sequences to the N-terminal portion of H-Ferritin subunits. In particular, two HFn constructs were designed inserting in the N-terminal position PAS sequences of different lengths: 40 (HFt-PAS40) and 75 (HFt-PAS75) amino acids, respectively. The PAS40 and PAS75 polypeptides were both genetically fused to HFn throughout a linker sequence consisting of three glycine residues that ensures proper PAS exposure on the outer surface of HFn. Both HFn mutants displayed high stability in plasma and outstanding efficiency in the encapsulation of doxorubicin. Indeed, the stability and circulation time of HFn-PAS-DOXO complexes were dramatically increased with respect to those of wild-type protein.^15^

Moreover, with the aim of increasing both half-life and tumor-targeting ability, H-Ferritin nanocages were genetically fused with the PAS sequence via two different linker sequences (GFLG and PLGLAG) and with the tumor-targeting peptide RGDK (Arg-Gly-Asp-Lys). In particular, GFLG and PLGLAG constitute cleavable sites that are recognized by cathepsin B and matrix metalloproteinase-2/9, respectively. The *in vivo* pharmacokinetics study revealed the positive impact of this strategy on HFn’s half-life. Indeed, approximately 4.9-fold longer circulation time was observed in comparison to the wild-type form, thus allowing enhanced retention time at the tumor site. The addition of RGDK, on the other hand, was successful in improving biodistribution, uptake efficiency, and targeting ability at the tumor site by specifically binding to integrin αvβ3/5 and neuropilin-1, which are expressed at high levels in different tumor types.^16^

In another work, a different variant of PASylated H-Ferritin nanocages was designed with the aim of preventing healthy cells’ internalization while ensuring specific tumor targeting. The authors genetically inserted, between the sequence encoding for H-Ferritin and the PAS sequence, a linker sequence named MP recognized by tumor matrix metalloproteases (MMPs). Thus, while the presence of the PAS shield promotes the extension of the *in vivo* stability, the recognition of the MP sequence by MMPs enables the unmasked HFn to freely interact with TfR1 overexpressed in cancer cells, triggering tumor-specific accumulation. Again, these H-Ferritin constructs displayed a longer half-life and greater drug encapsulation efficiency compared to the wild type HFn.^27^

**Figure 3:**
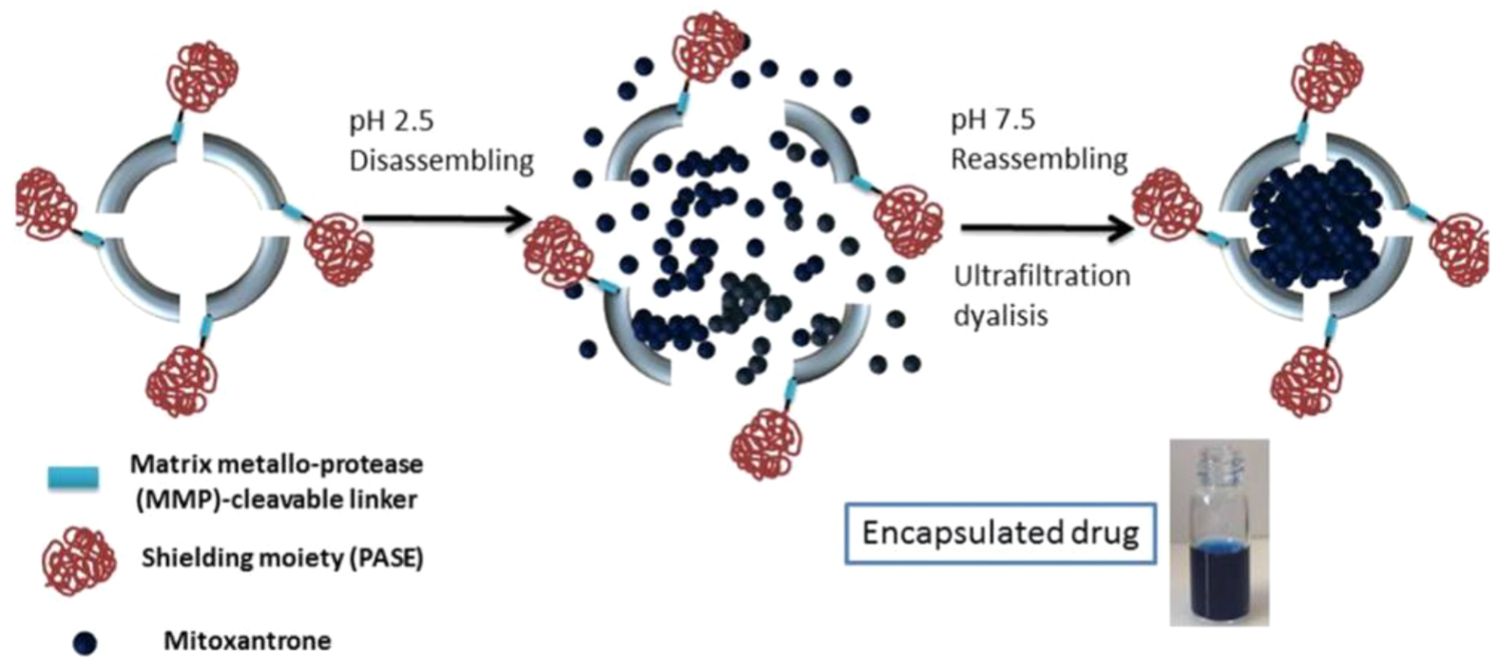
Scheme of HFn-PASE loading strategy. Reproduced with permission from ref (28). Copyright 2022 Elsevier B.V.

Based on the evidence that negatively charged nanocages can have different behaviors in terms of circulation time, HFn-MP-PAS was redesigned by adding two glutamic acid (E) residues, resulting in a new construct called HFn-MP-PASE. Through this modification, increased circulation time and longer accumulation at the tumor site of HFn nanocages were observed compared with the previously assessed nanocages thanks to the reduced undesired interaction with healthy tissues (Figure 3).^28^

Tesarova et al. proposed novel HFn-based nanoconstructs modified on the surface with PAS sequences of 10 and 20 amino acids, respectively. Here, to enable functionalization of the surface with PAS peptides, the surface of H-Ferritin nanocages was first decorated with gold nanoparticles, and subsequently PAS sequences were incubated, obtaining the final nanoconstruct. HFn nanocages modified with PAS10 and loaded with the cytostatic drug ellipticine displayed increased accumulation at the tumor, while its uptake into off-target tissues was hampered in a murine model of triple-negative breast cancer.^29^

### 5.2 Modification of H-Ferritin Nanocages with Albumin Binding Domain (ABD)

Another interesting modification that has been proposed to improve the performance of H-Ferritins as drug delivery vehicles involves their genetic functionalization with a variant of the ABD. This strategy is intended to exploit the high affinity that ABD has for human serum albumin (HSA), the most abundant protein found in plasma. Thus, by coating the outer surface of HFn nanocages with ABD, an increase in circulation time is expected to occur.

In order to study the pharmacokinetic profile *in vivo*, HFn nanocages were loaded with doxorubicin, and the half-life was evaluated by monitoring the drug’s concentration at different time points subsequent to the intravenous administration of ABD-HFn/DOX, HFn/DOX, and DOX. As a result, ABD-HFn/DOX displayed an extended half-life compared to both the free drug (19-fold longer) and HFn/DOX (12-fold longer). Furthermore, it was observed that ABD does not affect cellular uptake, as genetically modified nanocages showed results comparable to those obtained with wild-type HFn nanoparticles.^30^

### 5.3 XTENylation of H-Ferritin Nanocages

Another approach studied to mask the surface of nanocages with the aim of improving their stability is the method known as XTENylation. XTEN represents a class of unstructured polymers consisting of six repeating hydrophilic amino acids (A, E, G, P, S, and T) that can be genetically fused to obtain XTENylated proteins. The circulation time of XTEN polymers increases proportionally to their length since longer polypeptides confer larger hydrodynamic volumes, resulting in slower renal clearance. It has been observed that conjugation of XTEN polymers of the same length with therapeutic peptides or proteins can alter the half-life times of individual drugs differently. In any case, the half-life of each individual molecule can be altered depending on the length of the XTEN polymer.^17^

In another work, long-circulating ferritin nanocages (LCFNs) using intrinsically disordered proteins called “IDP cloud” were designed through 3D modeling with the purpose of shielding the nanoparticles and increasing the half-life time. Based on 3D modeling, ferritin monomers were genetically functionalized with XTEN polymers of different lengths (LCFN36, LCFN72, LCFN144, LCFN288), consisting of only hydrophilic amino acids (P, A, T, G, E, S), with the aim of understanding which length was optimal to give a longer half-life time. The results showed that increasing the length of the peptide achieved an increment in the half-life time compared to the wild-type form, thus showing a correlation between the two factors, while no significant difference was observed between LCFN144 and LCFN288.^21^

In addition, XTEN polymers have been observed to exhibit high biodegradability (thus avoiding accumulation in tissues following prolonged treatment), high bioavailability, and low or absent immunogenicity, making XTENylation a promising modification strategy.^17^

All significant efforts involving surface functionalization aimed at improving *in vivo* circulation time of ferritin nanocages, discussed herein, are summarized in Table 1.

**Table 1:** Summary of All Significant Efforts Involving Surface Functionalization Aimed at Improving *In Vivo* Circulation Time of Ferritin Nanocages

Modification Strategy	Material	Achievements	Ref
**Chemical Modifications**			
PEGylation	PEG sequences + α-MSH peptide	• Extended circulation time up to 24 h	(20)
		• Specific recognition and internalization into melanoma cells	

Clustering into oligomers	PEG sequences	• Prolonged circulation time and enhanced tumor uptake with the nanostructure consisting of 4 HFn monomers	(22)

Biomineralization	CaP shielding	• Increased *in vivo* half-life	(23)
		• Reduced uptake by liver cells	
		• Enhanced accumulation at the tumor	

**Genetical Modifications**			
PASylation	PAS sequence	• Enhanced stability in plasma	(15)
		• Improved encapsulation efficiency of doxorubicin	

	PAS sequence + RGDK targeting peptide	• Prolonged half-life (4.9-fold increase)	(16)
		• Improved tumor targeting ability	

	PAS sequence + cleavable linker recognized by tumor metalloproteases	• Extension of the *in vivo* stability promoted by PAS sequences	(27)
		• Specific tumor interaction thanks to the unmasking of PAS sequences at the tumor microenvironment	
		• Enhanced drug encapsulation efficiency	

	PASE sequence + cleavable linker recognized by tumor metalloproteases	• Increased circulation time thanks to the addition of acidic residues	(28)
		• Longer accumulation at the tumor site and reduced undesired interaction with healthy tissues	

	PAS sequence	• Increased accumulation at the tumor	(29)
		• Reduced uptake by healthy tissues	

Fusion with albumin binding domains	Coating with albumin	• Extended circulation time and improved pharmacokinetic profile	(30)

XTENylation	XTEN polypeptides	• Improved half-life in relation to the length of the XTEN polymer	(17)

	XTEN polypeptides	• Improved half-life in relation to the length of the XTEN polymer	(21)
		• High bioavailability and very low immunogenicity	

## 6 Conclusions

In the past decade, hundreds of nanodrug delivery systems based on HFn have been proposed. Several studies have demonstrated that ferritin nanocarriers can not only improve the bioavailability of soluble drugs and drive a specific accumulation at the tumor but also mitigate the side effects of toxic drugs on healthy tissues. However, some key challenges need to be addressed, including the relatively low stability and short *in vivo* half-life of ferritin. At present, different methods that include chemical and genetical modifications have been proposed as functionalization strategies to optimize the employment of ferritin nanosystems. Many of them hold great potential in tumor therapy and seem promising in tackling the major challenges described.

Clearly, considering the growing number of related publications, the PASylation strategy is one of the most compelling. Indeed, in the face of a relatively easy production, this functionalization strategy is reported to help overcome many of the current difficulties in the use of ferritin-based assemblies for *in vivo* applications. Overall, also biomineralization of HFn nanocages with calcium phosphate presents itself as a very new and promising strategy to tackle the above-discussed limitations and to advance toward the development of protein-based nanoplatforms for effective diagnostic and therapeutic applications.

In conclusion, further translational efforts based on ferritin nanoparticles as fine-tuned anti-tumor drug delivery platforms are expected in the near future.
